# Functional Polymer-Based Biomaterials for Corneal Tissue Engineering: An Indication-Guided Review of Design Trade-Offs

**DOI:** 10.3390/polym18192420

**Published:** 2026-10-04

**Authors:** Xinyue Zhang, Ruocheng Zhao, Yang Xu, Lei Sun, Yang Yang, Min Jiang

**Affiliations:** 1 State Key Laboratory of Materials-Oriented Chemical Engineering, College of Biotechnology and Pharmaceutical Engineering, Nanjing Tech University, Nanjing 211800, China; 2 Beijing Jishuitan Hospital, Capital Medical University, Beijing 100035, China

**Keywords:** corneal tissue engineering, functional polymers, polymeric biomaterials, polymer functionalization, hydrogels, corneal regeneration

## Abstract

Corneal disease is a cause of vision loss worldwide, while conventional keratoplasty is constrained by limited donor tissue and immune rejection. This indication-guided review examines functional polymer platforms for corneal tissue engineering, including natural polymers, synthetic polymer networks and scaffolds, and hybrid polymer composites. It examines how molecular composition, crosslinking, architecture, surface functionalization, and fabrication influence hydrogels, films, fibrous scaffolds, injectable matrices, delivery systems, and polymeric keratoprosthetic components for epithelial, stromal, and endothelial contexts. Using an indication-guided design trade-off framework, the review compares these systems across optical performance, mechanical performance and surgical handling, mass transport and degradation behavior, biological function and tissue integration, host response, and manufacturability. Natural polymers can provide biological cues that support cell interactions, with limitations in mechanical robustness and compositional consistency. Synthetic polymers offer greater control of network properties and processing but often require biofunctionalization to support tissue integration. Hybrid constructs can distribute optical, structural, and therapeutic functions across components or regions, with added fabrication complexity. No material class is universally optimal, and polymer selection should be guided by the target corneal layer and clinical indication. Standardized evaluation, reproducible reporting of polymer composition and fabrication conditions, scalable manufacturing, and transparent reporting of evidence maturity are priorities for clinical translation.

## 1. Introduction

The cornea is the transparent anterior refractive tissue of the eye. It provides approximately two thirds of the total refractive power of the eye and acts as a barrier against external pathogens and mechanical injury. Histologically, the cornea comprises five layers from anterior to posterior [1]. Bowman’s layer is an acellular anterior stromal layer distinct from the epithelial basement membrane, whereas Descemet’s membrane forms the basement membrane of the endothelium. The stroma accounts for approximately 90% of corneal thickness and contains orthogonally arranged lamellae of collagen fibrils with diameters of 25 to 35 nm. The regularity of fibril diameter and interfibrillar spacing is central to corneal transparency, while lamellar organization contributes to biomechanical stability [2].

A central transmittance of 87.1% at 500 nm was reported in ex vivo human corneas after normalization to a central thickness of 550 μm and adjustment to near-physiological hydration [3]. Optical comparisons should therefore specify wavelength, sample thickness, hydration, and measurement medium and distinguish single-wavelength values from spectral averages.

Penetrating and lamellar keratoplasty remain standard treatments for corneal blindness, but donor tissue availability remains far below the global demand for transplantation [4]. Donor shortage, immune rejection, variation in tissue quality, and high graft failure rates in high-risk eyes, including infected and neovascularized corneas, have driven the development of keratoprostheses and tissue-engineered corneal substitutes [5]. Biomaterial-based constructs offer routes for corneal reconstruction by reproducing selected extracellular matrix, structural, and functional features of native tissue.

Functional polymer platforms provide a controllable route to corneal reconstruction. Polymer chemistry, molecular weight, crosslinking, water content, network or scaffold architecture, surface functionalization, and fabrication can be adjusted to influence optical uniformity, surgical handling, molecular transport, degradation, cell interaction, and manufacturing reproducibility. Recent advances in hydrogel systems, tissue-derived matrices, polymer processing, and composite constructs have expanded the design space for corneal tissue engineering [6,7,8].

Because corneal layers have distinct repair requirements, functional polymer systems should be tailored to the target tissue and clinical indication [9]. Systems intended for ocular-surface and epithelial repair should promote epithelialization, control inflammation, and support tear-film stability [10]. Stromal constructs must maintain optical uniformity and regular curvature while providing mechanical support. Their optical and mechanical properties should therefore be assessed at the intended hydrated thickness [11]. Descemet’s membrane provides the native substrate for the endothelial monolayer. Endothelial cell carriers should therefore be thin and optically clear, withstand surgical insertion and unfolding, and support endothelial barrier and fluid-transport functions [12,13]. Full-thickness corneal substitutes may use core–skirt architectures to separate central optical function from peripheral fixation [14]. Candidate systems should therefore be evaluated across five interdependent requirements, including optical performance, mechanical performance and surgical handling, mass transport and degradation behavior, biological function and tissue integration, and manufacturability.

This review focuses on functional polymer systems for corneal tissue engineering. A functional polymer system is defined here as a natural, synthetic, or hybrid polymer whose molecular composition, chemical modification, crosslinking, architecture, surface treatment, or fabrication is deliberately used to control a function relevant to corneal repair. The scope includes hydrogels, films, fibrous scaffolds, injectable matrices, therapeutic delivery systems, and polymeric keratoprosthetic components when polymer design directly supports corneal repair, regeneration, optical restoration, cell delivery, therapeutic modulation, or tissue integration. Tissue-derived extracellular matrix constructs are considered as polymer-rich translational comparators. Particle-based systems are included when the polymer phase controls particle dispersion, release, structural retention, or optical behavior. Conventional devices are also included only as comparators for corneal protection, optical replacement, or tissue fixation. Routine refractive lenses, intraocular lenses, and delivery systems unrelated to corneal repair are excluded. Device retention or drug delivery alone is not considered evidence of tissue regeneration.

**Figure 1:**
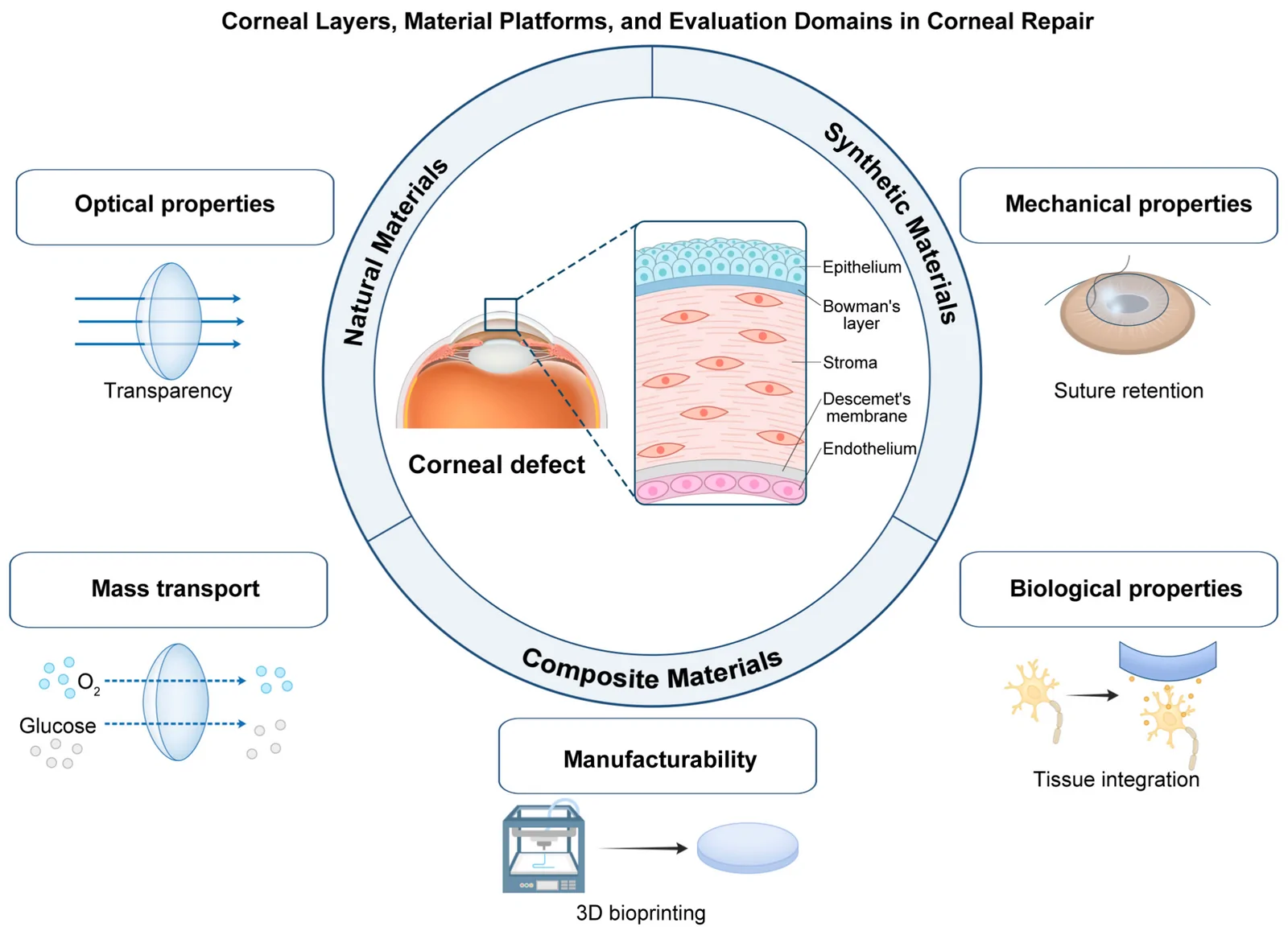
Overview of corneal layers, material platforms, and evaluation domains considered in this review. The schematic depicts the five-layer corneal anatomy together with natural, synthetic, and composite material platforms. Candidate systems are considered in relation to optical performance, mechanical performance and surgical handling, mass transport and degradation behavior, biological function and tissue integration, and manufacturability.

The five requirements are interdependent and create recurrent design tradeoffs among transparency and mechanical support, biointegration and long-term stability, and degradability and structural maintenance [15]. Starting with the target corneal layer and clinical indication, the framework identifies priority functional requirements and uses them to evaluate polymer chemistry, construct format, structural design, and fabrication route. Building on previous studies of natural biomaterials, hydrogels, and corneal regeneration, this review links material selection to clinical indications through explicit comparison of design trade-offs and evidence maturity. Figure 1 provides an overview of the corneal layers, material platforms, and evaluation domains considered in this review.

This narrative review was based on literature retrieved from Google Scholar using the keywords “corneal tissue engineering,” “functional polymers,” and “polymeric biomaterials.” The search primarily covered studies published from 2000 onward, with selected earlier publications included for their foundational relevance. Only English-language publications were considered. Additional studies were identified by screening the reference lists of relevant articles. References were organized and managed using EndNote. Articles were selected for their relevance to polymer-based corneal repair, with particular attention paid to how polymer composition, structure, and functionalization influence performance for specific corneal layers and clinical indications.

## 2. Bioactive Natural Polymer Platforms

**Figure 2:**
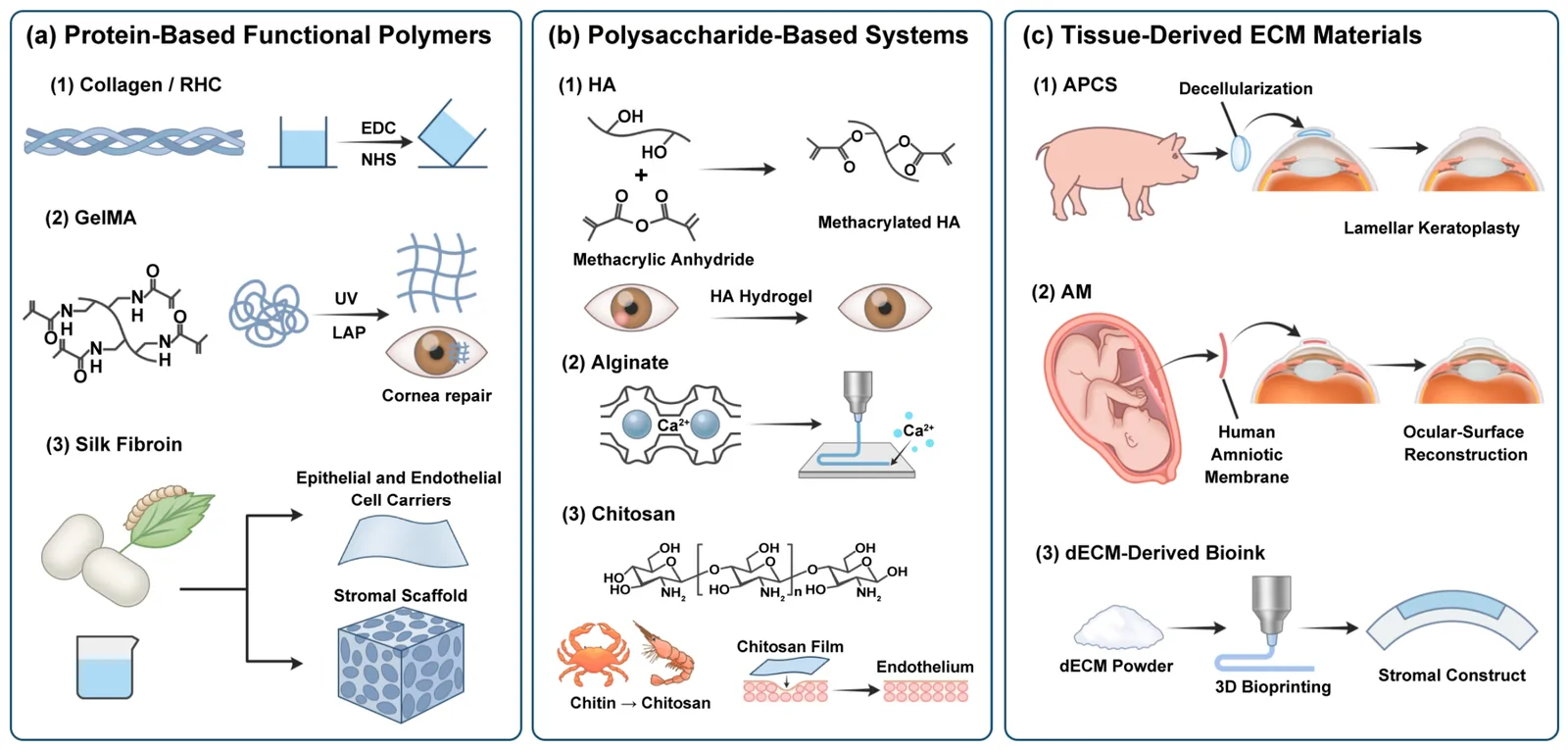
Natural polymer platforms and tissue-derived extracellular matrix materials for corneal repair. (**a**) Protein-based functional polymers, including collagen and recombinant human collagen (RHC), gelatin methacryloyl (GelMA), and silk fibroin. (**b**) Polysaccharide-based functional polymers, including hyaluronic acid (HA), alginate, and chitosan. (**c**) Tissue-derived extracellular matrix materials, including acellular porcine corneal stroma (APCS) for lamellar keratoplasty, amniotic membrane (AM) for ocular-surface reconstruction, and decellularized extracellular matrix (dECM)-derived bioinks for corneal stromal bioprinting. EDC denotes 1-ethyl-3-(3-dimethylaminopropyl)carbodiimide, NHS denotes N-hydroxysuccinimide, LAP denotes lithium phenyl-2,4,6-trimethylbenzoylphosphinate, and UV denotes ultraviolet light.

Bioactive natural polymer platforms are widely investigated for corneal tissue engineering because they provide hydrated, cell-interactive matrices and tissue-relevant biochemical cues [16,17]. Their molecular composition, chain interactions, crosslinking, and processing govern hydration, microstructure, degradation, and cell response, thereby determining their suitability for specific corneal indications. Figure 2 presents protein-based and polysaccharide-based functional polymers together with polymer-rich tissue-derived extracellular matrix materials, including decellularized corneal matrices, amniotic membrane (AM), and decellularized extracellular matrix (dECM)-derived bioinks. These platforms are evaluated according to polymer composition, microstructure, processing, and their capacity to meet indication-specific requirements for optical performance, mechanical performance and surgical handling, mass transport and degradation behavior, biological function and tissue integration, and manufacturability. Throughout this review, evidence is distinguished as laboratory characterization, in vitro cell studies, animal experiments, or human clinical evaluation, with each designation referring to the specific formulation and indication studied.

### 2.1. Protein-Based Functional Polymers

#### 2.1.1. Collagen- and Gelatin-Derived Functional Networks

Collagen is the main structural protein of the corneal stroma and accounts for approximately 70% of its dry weight. In the healthy stroma, type I collagen and type V collagen form the predominant fibrillar network, whereas type III collagen is more closely associated with stromal repair and fibrosis. The ordered arrangement of collagen fibrils and lamellae provides a structural basis for corneal transparency and serves as a reference for stromal substitute design [18]. Native collagen and recombinant human collagen (RHC) have therefore been explored for stromal replacement and intrastromal repair. Early chemically crosslinked collagen hydrogels functioned as implantable corneal substitutes, and collagen–chondroitin sulfate constructs supported three-dimensional corneal equivalents with morphology and transparency resembling native tissue [19,20].

RHC provides a defined amino acid sequence and more consistent production than animal-derived collagen. Recombinant human type III collagen (RHC-III) has been widely used in biosynthetic corneal implants because its crosslinking and gel formation can be controlled during manufacture. EDC/NHS crosslinking enables adjustment of the mechanical properties and degradation behavior of RHC-III implants through changes in formulation and crosslinking conditions [21,22]. In a clinical study, cell-free RHC-III implants integrated with host tissue, no clinical rejection was reported over four years, and the implants were accompanied by stromal cell and corneal nerve repopulation [23].

Mechanical weakness and limited suture retention remain important constraints for collagen-based implants. Increasing crosslink density, introducing secondary networks or peptide amphiphiles, and using in situ crosslinkable formulations can improve structural integrity and delay degradation. These modifications may also affect optical clarity, nutrient transport, and host-mediated remodeling, creating a design-tradeoff for stromal applications. In situ-forming collagen hydrogels crosslinked with multifunctional poly(ethylene glycol) have been investigated as matrix therapies for corneal defects [24,25]. A visible-light curable type I collagen bioadhesive crosslinked with 3,4 dihydroxyphenylalanine modified poly(ethylene glycol) diacrylate was developed for corneal perforation repair and showed strong wet tissue adhesion and favorable corneal keratocyte cytocompatibility in vitro [26]. A double-crosslinked bioengineered porcine collagen construct, BPCDX, increased stromal thickness, flattened corneal curvature, and improved visual function after intrastromal implantation in two clinical cohorts with advanced keratoconus [27].

Collagen MPC interpenetrating polymer networks can improve resistance to collagenase-mediated degradation while retaining favorable optical properties [28]. Separately, crosslinked MPC hydrogels have been evaluated for protein-adsorption resistance and oxygen permeability [29]. RHC-III implants have also shown host cell and nerve ingrowth with stable integration at 24 months [30]. Collagen and RHC systems are most relevant to stromal repair, where optical clarity, surgical handling, structural persistence, nutrient transport, and host remodeling must be considered together. RHC is particularly useful when a defined composition and reproducible manufacture are required.

Gelatin is a denatured derivative of collagen with documented ophthalmic biocompatibility [31]. Methacrylation of gelatin amine and hydroxyl groups yields gelatin methacryloyl (GelMA), a photocrosslinkable hydrogel widely used in tissue engineering and bioprinting. By varying the degree of methacrylation, polymer concentration, photoinitiator type and concentration, and light dose, the stiffness, swelling, degradation, and mesh size of GelMA hydrogels can be tailored [32]. These parameters collectively influence optical transparency, solute transport, cell viability, and printing fidelity. GelMA also retains cell-interactive motifs, including arginine–glycine–aspartic acid (RGD)-containing sequences, which support cell adhesion and spreading [33]. GelMA can serve as both a cell-laden stromal bioink and an in situ photocrosslinkable bioadhesive. GelMA hydrogels containing corneal stromal cells can maintain high optical transmittance while supporting cell survival and the expression of stromal phenotype-associated markers [34]. GelCORE is a visible-light-crosslinked gelatin-based bioadhesive that enabled sutureless closure of stromal defects and supported subsequent re-epithelialization and stromal regeneration in a rabbit corneal model [35]. Recent corneal bioprinting studies indicate that printable GelMA-based formulations can provide measurable optical transparency while supporting corneal stromal cell adhesion, proliferation, and stromal phenotype-associated marker expression. For example, a GelMA/methylcellulose construct achieved approximately 78% transmittance at 700 nm in an in vitro study [36]. In addition, digital light processing (DLP)-printed GelMA-based bioinks have enabled the programmable construction of corneal curvature while maintaining high cell viability and tunable mechanical properties [37].

The clinical translation of GelMA is primarily constrained by the need to balance mechanical stability and printability with optical clarity, mass transport, and cytocompatibility. Increasing polymer concentration or crosslink density can enhance structural integrity and delay degradation. However, these changes may also reduce nutrient diffusion, impair cell-mediated matrix remodeling, and increase the risk of phototoxicity [32,33]. Accordingly, secondary crosslinking and composite formulations are often used to improve the mechanical performance and printability of GelMA, although the safety of photoinitiators, residual crosslinking reagents, and degradation products requires careful evaluation [35]. GelMA is primarily investigated for corneal stromal engineering, corneal defect repair, and bioprinting, where print fidelity, optical transmittance, corneal stromal cell viability, and stromal phenotype are common evaluation endpoints. Current evidence remains predominantly derived from in vitro studies and preclinical animal models [36]. Its crosslinking process must provide adequate mechanical stability without compromising optical clarity, mass transport, cytocompatibility, or formulation reproducibility [37].

#### 2.1.2. Silk Fibroin-Based Functional Films and Scaffolds

Silk fibroin is a natural protein that can be processed into transparent films and three-dimensional scaffolds for corneal applications. In Bombyx mori, fibroin contains heavy and light chains linked by a disulfide bond [38]. Processing conditions regulate Silk II β-sheet crystallinity and thereby influence mechanical properties, structural stability, and degradation behavior while permitting the fabrication of transparent films and three-dimensional constructs [38]. After degumming, silk fibroin generally shows good biocompatibility and low immunogenicity, whereas residual sericin and processing can alter local inflammatory and foreign-body responses. Its cell response and interface behavior are influenced by silk source, residual sericin, processing conditions, and implantation site. Surface microtopography, multilayer assembly, and pore architecture provide additional control over cell alignment and material performance.

Regenerated silk fibroin can contain chains and aggregates in different conformational states, which alter the accessibility of reactive groups and complicate chemical functionalization. Controlling silk conformation and aggregation has been shown to improve the reproducibility of chemical modification and enable multiple modifications [39]. Integration of silk nanofiber assembly with microfluidic spinning can produce aligned hierarchical fibers that guide cell orientation and migration [40]. Together, these studies identify conformational control and hierarchical assembly as complementary design tools. For corneal applications, these processing choices should be evaluated alongside transparency at the intended construct thickness.

Thin silk fibroin films have been developed as transparent cell carriers, whereas multilayered and three-dimensional scaffolds can provide structural support for stromal repair. Micropatterned silk fibroin films with a thickness of approximately 2 μm guided cellular alignment and reproduced the lamellar length scale of the corneal stroma [41]. RGD functionalization and helically stacked multilayer structures have also been used to create transparent anisotropic stromal models [42]. Silk fibroin films support corneal endothelial cell adhesion, monolayer formation, and endothelial-associated functions [43]. Non-mulberry silk fibroin films showed visible-light transmittance above 95% and a refractive index comparable to that of the native cornea in vitro, while differences among silk varieties affected cell adhesion and degradation [43]. Silk fibroin endothelial grafts have also supported tissue integration and restoration of corneal transparency in a rabbit DMEK model [44]. Porous silk fibroin films have supported cultivated corneal epithelial sheets [45], while silk fibroin membranes also supported human corneal endothelial cell growth in vitro [46]. Silk fibroin–chitosan scaffolds have been investigated for stromal reconstruction [47].

The design requirements for silk fibroin depend on the target corneal layer. For stromal repair, increasing β-sheet crystallinity improves scaffold stability and slows degradation, although prolonged material persistence may restrict host–cell-mediated remodeling [48]. For endothelial delivery, films must remain optically clear and sufficiently thin while retaining adequate interfacial smoothness and resistance to postoperative folding. Silk fibroin films have supported corneal endothelial cells in vitro [49], and an aloe vera silk fibroin film showed short-term tissue integration without evident inflammatory reactions after transplantation in rabbit eyes [50]. Silk fibroin can therefore be adapted for epithelial, stromal, and endothelial applications through control of crystallinity, surface features, and construct architecture. Reproducible use requires control of silk source, residual sericin, and processing conditions, together with long-term assessment of degradation, interface behavior, and visual quality [51].

### 2.2. Polysaccharide-Based Functional Polymers

Polysaccharides provide hydrophilicity, biocompatibility, and chemical versatility for corneal tissue engineering. Their molecular composition, crosslinking chemistry, and processing determine hydration, structural retention, cell interaction, and mass transport, which in turn influence their use in different corneal indications. Chitosan is produced by the partial deacetylation of chitin, and its physicochemical properties, antibacterial activity, and adhesiveness vary with the degree of deacetylation and molecular weight [52,53]. Chitosan films have been explored for ocular-surface repair and corneal endothelial cell delivery. Their wettability, antimicrobial activity, transparency, and cytocompatibility are useful for these applications, whereas limited cell adhesion often necessitates surface modification or incorporation of cell-adhesive components [54]. Chitosan/PCL blend membranes supported bovine corneal endothelial cell proliferation, monolayer formation, and expression of ZO-1 and Na^+^/K^+^-ATPase in vitro. Chitosan processing is particularly sensitive to pH because its amino groups are protonatable. Acid-solubilized formulations must retain optical uniformity and adequate wet strength after neutralization and hydration, while surface modification should improve cell attachment without compromising transparency [55].

Hyaluronic acid (HA) is widely used in ocular-surface formulations, while functionalized HA hydrogels are being investigated for sutureless repair of corneal defects. Its high water-retention capacity, viscoelasticity, and adhesiveness can promote corneal epithelial migration and regulate cell behavior and inflammatory responses through cluster of differentiation 44 (CD44)-associated mechanisms [56,57,58]. Methacrylation and dynamic covalent crosslinking increase the structural stability of HA hydrogels for wound coverage, cell delivery, and in situ repair [59,60,61]. Increasing network density improves stability but can restrict solute diffusion and cell migration. HA-based bioinks have also been used to construct cell-laden corneal stromal models with potential for shape fidelity and tissue integration [62]. HA formulations should therefore be matched to the required duration of wound coverage or cell delivery while preserving transparency, transport, and timely remodeling.

Alginate is commonly used as a stromal bioink and cell-encapsulation matrix. Calcium ions interact with guluronic acid blocks to form an egg-box ionic network, allowing rapid gelation under mild conditions [63]. Keratocytes encapsulated in alginate-based bioinks retained high viability immediately after printing and after 7 days of culture [64]. The ionic network can weaken through ion exchange, and alginate lacks cell-adhesive motifs. Adhesive components and secondary crosslinking are therefore used to improve cell interaction and structural retention [65]. For stromal bioinks, these modifications should preserve optical uniformity, nutrient transport, degradation control, and printing reproducibility.

Other polysaccharides provide additional options for specific applications. Oxidized dextran can be crosslinked with gelatin to form injectable hydrogels for alkali-burn corneal repair [66]. Bacterial cellulose has high purity, a nanofibrillar network, and favorable optical transparency, supporting its development as a corneal dressing and scaffold for epithelial regeneration [67].

### 2.3. Polymer-Rich Tissue-Derived Extracellular Matrix Materials

Tissue-derived extracellular matrix (ECM) materials provide polymer-rich translational comparators for corneal tissue engineering. Acellular porcine corneal stroma (APCS), amniotic membrane (AM), and decellularized ECM (dECM)-derived bioinks retain tissue-specific matrix components and structural features that remain difficult to reproduce in compositionally defined polymer systems. For decellularized matrices, the preparation method determines the extent of cell removal, as well as the preservation of native architecture, mechanical integrity, and host integration [68,69].

APCS is mainly used as a lamellar graft for stromal replacement. Its size, thickness, and lamellar stromal organization resemble those of the human cornea, and it has been used in lamellar keratoplasty for infectious corneal ulcers, including fungal keratitis [70,71]. Crosslinking improves surgical handling and resistance to enzymatic degradation [72]. Material processing also affects epithelial healing, long-term transparency, degradation, and the risk of postoperative calcification [73]. APCS is therefore most relevant when stromal structure and graft handling are the main clinical requirements.

AM is widely used for ocular-surface reconstruction in persistent epithelial defects, limbal stem cell deficiency (LSCD), and chemical burns. Its basement membrane contains type IV collagen and laminin, which support epithelial cell adhesion and migration and contribute to the regulation of inflammation, fibrosis, and neovascularization [74,75,76]. AM is commonly used as a wound covering for ocular-surface repair [77,78], and denuded AM can serve as a carrier for ex vivo expanded limbal epithelial cell sheets in LSCD treatment [79]. Tissue source and preservation method influence its clinical performance [80]. Its primary role is to support epithelial healing and ocular-surface reconstruction rather than to provide load-bearing stromal replacement.

Decellularized corneal matrices can be processed into dECM powder and formulated as printable bioinks for stromal engineering [81]. These bioinks introduce cornea-specific matrix cues into cell-laden constructs, while their optical uniformity, structural stability, and batch consistency depend on formulation and process control. APCS, AM, and dECM bioinks therefore address different clinical needs, including stromal replacement, ocular-surface reconstruction, and stromal cell delivery. They also show how tissue source, decellularization, crosslinking, and processing influence the balance among optical performance, surgical handling, tissue integration, and manufacturability. Table 1 summarizes natural functional polymer systems and tissue-derived extracellular matrix materials for corneal repair.

## 3. Synthetic Functional Polymer Platforms

Synthetic functional polymers allow precise control of composition, architecture, and processing for corneal constructs. Molecular weight, monomer composition, crosslink density, hydrophilic and hydrophobic balance, and network architecture can be adjusted to control optical clarity, hydration, mechanical behavior, mass transport, and, where relevant, degradation kinetics [82]. This controllability supports reproducible manufacture and indication-specific design. The limited cell recognition of many synthetic polymers makes surface functionalization, bioactive ligand incorporation, or combination with natural components important for tissue integration. Permanent devices also require long-term assessment of device-specific interface complications. In PHEMA-based AlphaCor keratoprostheses, reported concerns include stromal melt, retention-related complications, and deposits [83,84,85]. PMMA-based Boston keratoprostheses require separate monitoring of long-term retention, tissue melting, infection, glaucoma, and retroprosthetic membrane formation [86,87,88].

**Figure 3:**
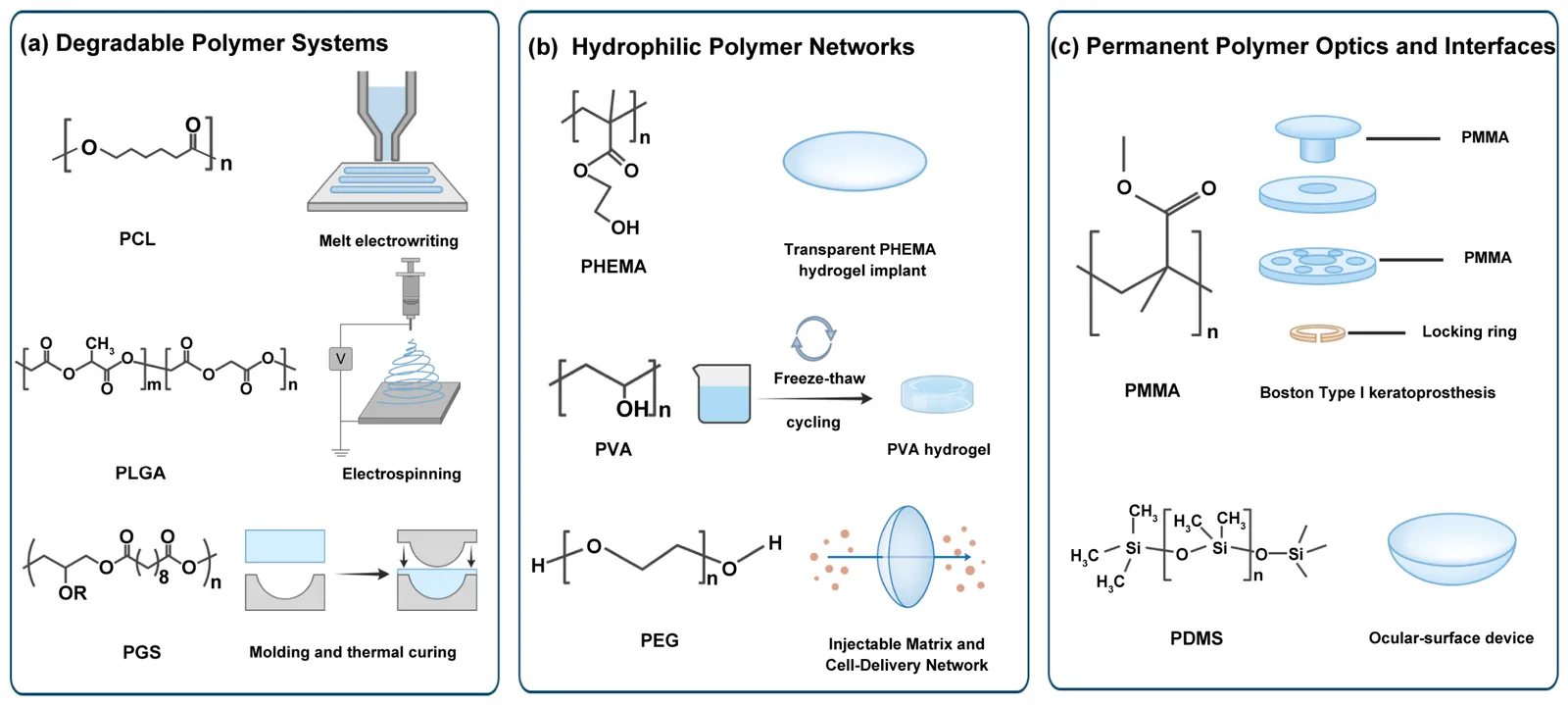
Synthetic functional polymer systems, design strategies, and corneal applications. (**a**) Degradable polymer systems based on poly(caprolactone) (PCL), poly(lactic-co-glycolic acid) (PLGA), and poly(glycerol sebacate) (PGS). Representative processing routes include melt electrowriting for PCL, electrospinning for PLGA, and molding followed by thermal curing for PGS. (**b**) Hydrophilic polymer networks, including a transparent poly(2-hydroxyethyl methacrylate) (PHEMA) implant, poly(vinyl alcohol) (PVA) hydrogels formed by freeze–thaw cycling, and poly(ethylene glycol) (PEG)-based networks for injectable matrices and cell-delivery platforms with tunable solute transport. (**c**) Permanent polymer optics and interfaces, including poly(methyl methacrylate) (PMMA) keratoprosthetic optics and poly(dimethylsiloxane) (PDMS) ocular-surface interfaces.

This review considers degradable scaffolds, stable hydrophilic networks, and permanent polymer components as distinct design formats. Poly(caprolactone) (PCL), poly(lactic-co-glycolic acid) (PLGA), and poly(glycerol sebacate) (PGS) provide temporary structural support and are designed to resorb during tissue repair. Poly(2-hydroxyethyl methacrylate) (PHEMA), silicone hydrogels, poly(vinyl alcohol) (PVA), and selected poly(ethylene glycol) (PEG) networks provide stable hydrated interfaces with controllable water content, transport, and mechanical behavior. Poly(methyl methacrylate) (PMMA) and poly(dimethylsiloxane) (PDMS) are used in durable optical or interfacial components when the clinical indication requires long-term structural stability. Figure 3 presents these material systems in relation to their processing routes and corneal applications.

### 3.1. Biodegradable Synthetic Polymer Scaffolds

Degradable synthetic polymers provide temporary structural templates that support cell migration, matrix deposition, and tissue remodeling. Processing routes depend on polymer chemistry, as thermoplastic polyesters can form fibrous scaffolds, whereas conventional PGS is shaped from a prepolymer and thermally crosslinked. For corneal repair, degradation kinetics and the decline in scaffold support need to be coordinated with new tissue formation [89].

PCL is primarily investigated as a fibrous scaffold for corneal stromal engineering. Its slow resorption prolongs the material–tissue interface and may extend exposure to foreign-body and fibrosis-related responses, whereas thermal processability and the capacity to form aligned or layered fibers support architectures that guide keratocyte alignment and provide mechanical reinforcement [90,91]. Fiber diameter, scaffold thickness, packing density, and refractive-index matching influence light scattering and optical uniformity. PCL design therefore requires a balance between aligned mechanical guidance, transparency, and timely host-mediated remodeling.

PLGA can be processed into porous membranes or fibrous scaffolds for temporary epithelial or endothelial cell delivery. The lactide-to-glycolide ratio, molecular weight, and scaffold architecture regulate degradation and support duration [92,93]. Its degradation may generate acidic products. In a non-ocular in vitro model, these products promoted pro-inflammatory macrophage responses [94], making local pH control and tissue compatibility important design considerations. PLGA is most applicable to temporary delivery formats that require short-term structural support and controlled resorption.

PGS is a degradable elastomer whose mechanical behavior can be adjusted through polymer composition and curing conditions. Conventional PGS requires thermal curing before cell incorporation, and progressive erosion reduces its load-bearing cross-section. Its potential use as a compliant corneal support therefore depends on maintaining geometry and hydrated transparency during degradation; direct corneal validation remains limited [95].

PCL is suited to stromal applications requiring persistent aligned support, whereas PLGA is more applicable to temporary epithelial or endothelial delivery. PGS provides a compliant degradable option when temporary mechanical support is needed. Selection among these systems should be guided by the target corneal layer, required residence time, optical requirements, and tissue-integration needs.

### 3.2. Stable Polymer Networks and Device Components

#### 3.2.1. Hydrophilic Functional Polymer Networks

Hydrophilic synthetic polymers form hydrated, three-dimensional networks that combine optical clarity with water and solute transport. Network persistence and degradation depend on polymer chemistry and crosslink design. Polymer composition, water content, crosslink density, and network architecture govern optical behavior, mechanical properties, and diffusion. Because many synthetic networks provide limited cell-recognition signals, formulation, surface modification, and crosslinking should be selected according to the intended tissue interface.

PHEMA is an established hydrogel material for keratoprosthesis design. Crosslinked PHEMA forms a transparent, water-insoluble, and highly hydrated network. AlphaCor comprises a transparent central optic and a porous peripheral skirt based on PHEMA, joined through an interpenetrating network region. The porous skirt supports cellular ingrowth and tissue integration [96]. The device was developed for corneal replacement in patients with a poor prognosis for conventional keratoplasty. Key evaluation endpoints include optical clarity; tissue integration; device retention; stromal melt; retroprosthetic membrane formation, which is a fibrosis-related interface outcome distinct from graft-type immune rejection; and optic deposition. Calcium and pigment deposition can be influenced by topical medications and patient-related factors, making long-term optical stability an important consideration. PHEMA is therefore relevant to keratoprosthetic applications requiring a stable optical zone and peripheral tissue integration, while cell-mediated remodeling remains limited.

Silicone hydrogels are nondegradable polymer networks used mainly in therapeutic bandage contact lenses and ocular-surface protection. Their siloxane components increase oxygen permeability while preserving hydration, supporting short-term coverage after surgery or ocular-surface injury. Oxygen permeability, epithelial recovery, wettability, and wear safety are key evaluation parameters [97,98,99]. Tear-film instability, mechanical friction, and infection risk remain important determinants of clinical safety.

PVA hydrogels are generally nondegradable under corneal-use conditions and are mainly investigated as contact-lens materials, with exploratory applications in stromal membranes. Freeze–thaw cycling produces physically crosslinked PVA networks without residual chemical crosslinkers. Polymer concentration and freeze–thaw conditions regulate optical clarity, water content, and mechanical properties [100]. Increasing the number of freeze–thaw cycles improves network stability and can reduce water content and solute transport. Current corneal evidence is predominantly preclinical, and long-term stability requires further evaluation [101]. The principal design challenge is to control crystallization and swelling so that improved wet strength does not introduce optically heterogeneous domains or restrict solute transport [102].

PEG is a hydrophilic synthetic polymer used to construct crosslinked hydrogel networks. Molecular weight, polymer concentration, crosslink chemistry, and network architecture regulate mechanical properties and mass transport. In long-persistence PEG networks, degradation is determined primarily by crosslinker chemistry and incorporated labile segments. PEG-based systems are widely used as injectable matrices and cell-delivery platforms. Bioactive ligand incorporation, surface modification, or hybrid network design is important when cell adhesion and tissue integration are required. Adhesive ligands provide cell-binding sites, whereas degradable crosslinks create space for cell spreading and matrix deposition. These functions need to be designed together. Excessive network persistence can restrict remodeling, while rapid cleavage can compromise shape retention before new tissue provides support [103].

These materials serve distinct clinical indications. PHEMA supports permanent keratoprosthetic optical replacement and peripheral fixation. Silicone hydrogels support oxygen-permeable short-term ocular-surface protection. PVA provides a transparent physically crosslinked platform with adjustable hydration and mechanics. PEG enables injectable and cell-delivery networks with controlled transport and mechanical properties. Material selection should be guided by the target corneal layer, clinical indication, and the required balance among optical performance, mechanical handling, mass transport, tissue integration, and long-term stability.

#### 3.2.2. Permanent Polymer Optics and Interfaces

Permanent synthetic polymers are used mainly as keratoprosthetic components and ocular-surface interfaces because they provide optical stability, mechanical integrity, and reproducible manufacturing. Precision machining, compression molding, and injection molding can produce optical components and support structures with stable geometries. Low water content and limited intrinsic tissue interactions influence aqueous nutrient transport, cell adhesion, and host integration. Their design therefore requires long-term material stability together with appropriate interface engineering.

PMMA is widely used as the central optical cylinder of full-thickness keratoprostheses. As an amorphous thermoplastic, it provides favorable optical properties, chemical stability, and precise manufacturability for devices such as the Boston keratoprosthesis. Key clinical endpoints include long-term optical function, device retention, glaucoma, and retroprosthetic membrane formation. Long-term follow-up indicates that the Boston Type I keratoprosthesis can restore useful vision in eyes with a high risk of failure after conventional keratoplasty. Sustained clinical benefit depends on continued monitoring and management of postoperative complications.

PDMS is investigated mainly for ocular-surface interfaces and keratoprosthesis-related devices. Its flexible siloxane backbone and high oxygen permeability support compliant device interfaces. The methyl-rich hydrophobic surface can impair tear-film spreading and cell adhesion, while aqueous-solute transport remains limited. Oxygen plasma treatment improves surface wettability, and substrate stiffness influences epithelial cell spreading and proliferation [104,105]. PDMS design for corneal applications should therefore combine surface modification with control of interface stability and ocular-surface compatibility.

PMMA and PDMS occupy distinct roles among permanent polymer components. PMMA provides a durable central optical component for keratoprosthetic applications [86,87,88], whereas PDMS has been investigated as a compliant and surface-modifiable interface for ocular-surface and keratoprosthesis-related devices. These materials are therefore included as functional device components and interfacial materials. Their selection depends on whether the clinical indication requires durable optical replacement, mechanical fixation, or a flexible interface.

Across synthetic polymer platforms, degradable scaffolds, long-persistence hydrogel networks, and permanent device components address different indication-specific requirements. Selection should be guided by the relative priorities of optical function, mechanical support, mass transport, biological integration, and manufacturability. Hybrid and composite systems can combine structural, biological, and therapeutic functions within a single construct. Table 2 summarizes synthetic functional polymer systems for corneal repair.

## 4. Hybrid and Composite Functional Polymer Systems

Composite systems combine two or more polymeric, biological, or therapeutic components and distribute functions across molecular, fibrous, and regional structural scales. Natural–synthetic combinations can couple biological signals from natural materials with the controllable mechanics and processing properties of synthetic polymers. Component ratio, interfacial bonding, porosity, fiber alignment, and regional architecture influence optical clarity, mechanical support, mass transport, tissue integration, and long-term stability. In corneal repair, composite designs allocate these requirements across components or spatial regions according to the target layer and clinical indication [106].

**Figure 4:**
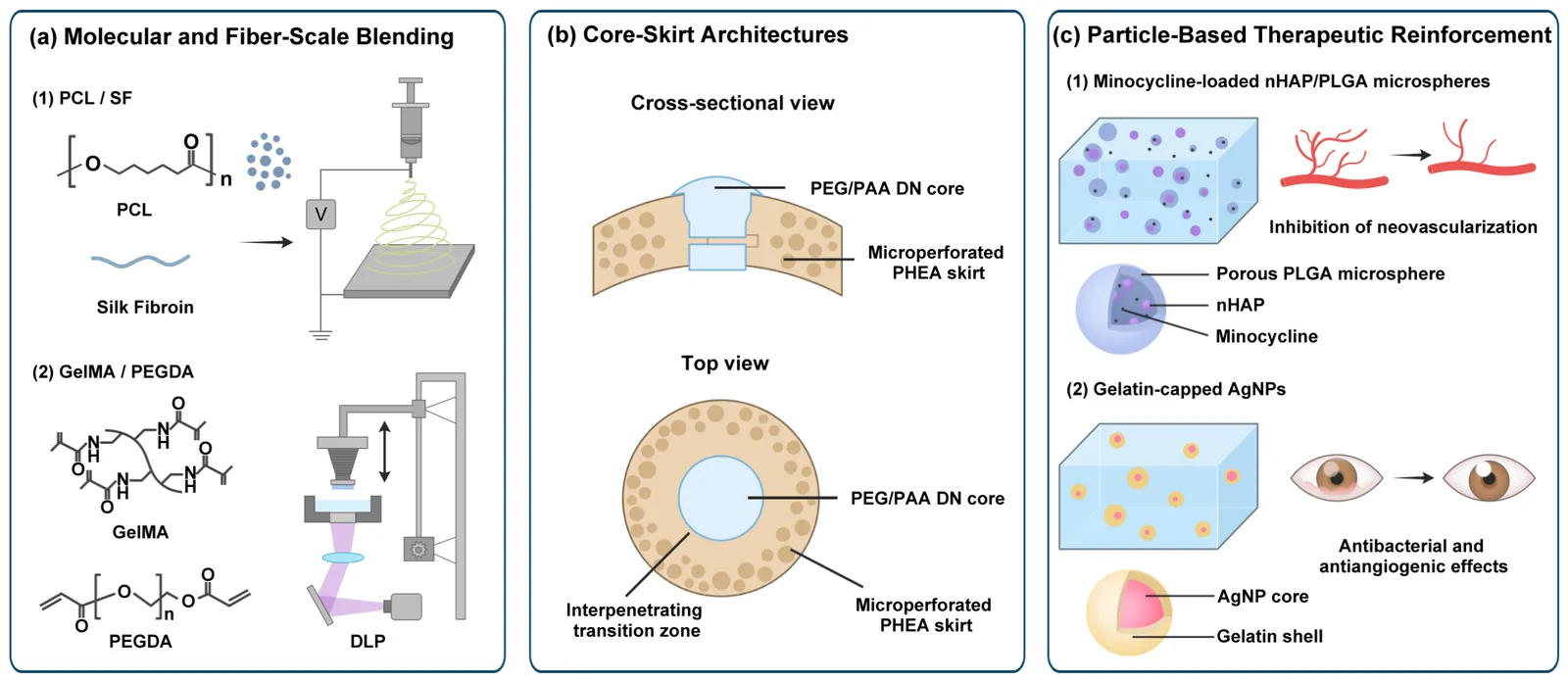
Composite functional polymer design strategies for corneal repair. (**a**) Molecular and fiber-scale blending, including electrospun poly(caprolactone) (PCL)/silk fibroin (SF) fibrous scaffolds and digital light processing (DLP)-printed gelatin methacryloyl (GelMA)/poly(ethylene glycol) diacrylate (PEGDA) constructs. (**b**) Core–skirt architecture comprising a poly(ethylene glycol)/poly(acrylic acid) (PEG/PAA) double-network (DN) hydrogel core and a microperforated poly(2-hydroxyethyl acrylate) (PHEA) hydrogel skirt joined through an interpenetrating transition zone. The PEG/PAA DN core supports the central optical zone, and the microperforated PHEA skirt supports peripheral tissue integration. Both core and skirt surfaces were functionalized with type I collagen. (**c**) Particle-based therapeutic and reinforcement systems, including minocycline-loaded nano-hydroxyapatite/poly(lactic-co-glycolic acid) (nHAP/PLGA) microspheres for corneal neovascularization and gelatin-capped silver nanoparticles (AgNPs) for antibacterial and antiangiogenic treatment of bacterial keratitis.

This section examines molecular blending, core–skirt architectures, and particle-containing composite systems. Figure 4 presents representative material combinations, structural formats, and corneal applications. Molecular blending can generate fibrous scaffolds and photocurable networks, including PCL/silk fibroin and gelatin methacryloyl (GelMA)/poly(ethylene glycol) diacrylate (PEGDA) systems. Core–skirt architectures combine a transparent central optic with a peripheral skirt that supports tissue integration and fixation. Nanoparticle- and microsphere-containing systems introduce bioactive or therapeutic components that regulate neovascularization, growth-factor presentation, inflammation, and corneal wound healing.

### 4.1. Molecular and Fibrous Polymer Blends

Molecular and fibrous blending is used primarily for corneal stromal regeneration, with selected constructs explored for ocular-surface support. Natural components provide cell-interactive cues, whereas synthetic components contribute structural stability and processing control. Component dispersion, interfacial bonding, and refractive-index compatibility influence optical performance, mechanical behavior, and tissue response. Phase separation and large fiber diameters can increase light scattering. Nonuniform component distribution and weak interfacial bonding can reduce structural integrity.

Fiber-containing blends provide contact guidance and mechanical support for stromal cells. Electrospun PCL/silk fibroin scaffolds show favorable fiber alignment, wettability, and cytocompatibility. At selected compositions, their tensile properties fall within the range reported for corneal tissue-engineering applications and support keratocyte attachment and proliferation in vitro [107]. Sandwich scaffolds composed of plastically compressed collagen and laser-perforated PLGA fibrous membranes combine a cell-supportive collagen matrix with a porous membrane that improves handling and suture retention. Laser perforation reduces optical loss while preserving structural support [108]. Layered composites containing electrospun PCL microfibers and gelatin glycidyl methacrylate (G-GMA) hydrogels improve mechanical performance and suture retention while supporting corneal cell migration, proliferation, and phenotypic maintenance [109].

Hydrogel blends support cell function and structural retention in hydrated constructs. In photocrosslinked GelMA/PEGDA systems, PEGDA increases the mechanical stability and print fidelity of GelMA constructs while maintaining cell adhesion, nutrient transport, and optical transmission. DLP-printed GelMA/PEGDA bilayer implants promoted re-epithelialization and stromal repair in a rabbit anterior lamellar keratoplasty model. Across the PEGDA-GelMA-10-5, -15-5, and -20-5 formulations, transmittance at 600 nm decreased from 90.7% to 86.3% and 82.5%, respectively, versus 77.9% for the native-cornea control (spectral scan, 400–800 nm) [110]. GelMA-pHEMA interpenetrating polymer network hydrogels increased the compressive modulus of corneal stromal models in vitro while maintaining keratocyte viability, extracellular-matrix deposition of collagens and proteoglycans, and high optical transparency [111]. Cell-laden GelMA incorporated into decellularized corneal matrix improved optical transmission and partially restored mechanical performance while retaining cornea-derived extracellular-matrix cues. Component ratio and crosslinking conditions govern structural retention, mass transport, and cell-mediated remodeling in these constructs [112].

Fiber-containing blends are suited to stromal repair requiring contact guidance and surgical handling. Fiber size, spacing, and component dispersion should preserve optical uniformity and solute transport. Hydrogel blends are applicable when hydration, optical transmission, and cell function are central requirements. Crosslink density and component ratio should provide adequate structural retention while preserving diffusion and remodeling. Current evidence is derived mainly from in vitro studies and animal models. Long-term evaluation should address optical clarity, interface stability, degradation behavior, and tissue integration.

### 4.2. Core–Skirt Architectures

Core–skirt architectures are designed for full-thickness keratoprosthetic replacement in high-risk eyes. They separate a transparent central optic from a peripheral skirt engineered for fixation and tissue integration. This regional organization assigns optical and anchoring functions to different parts of the construct and supports long-term implant stability [113].

Autologous tissue-based fixation is represented by the osteo-odonto-keratoprosthesis (OOKP), which serves as a clinical comparator for core–skirt designs. In OOKP, a PMMA optical cylinder is secured within an autologous tooth-alveolar bone lamina and covered with oral mucosa to establish biological anchorage. The procedure is used mainly in patients with end-stage ocular-surface disease, severe dry eye, chemical burns, or repeated corneal graft failure. Long-term use requires management of glaucoma, vitreous hemorrhage, mucosal overgrowth or ulceration, and retroprosthetic membrane formation [114,115].

Hydrogel-based core–skirt systems use regional variation in polymer composition and porosity to support tissue integration. Early PHEMA core–skirt keratoprostheses combined a transparent optic with a porous skirt that supported fibrovascular ingrowth in rabbits [14]. A photolithographically fabricated construct used a poly(ethylene glycol)/poly(acrylic acid) (PEG/PAA) double-network hydrogel as the central optic and a type I collagen-modified, microperforated poly(2-hydroxyethyl acrylate) (PHEA) hydrogel as the peripheral skirt. The two regions were joined through an interpenetrating zone. Both core and skirt surfaces were functionalized with type I collagen. In vitro, the core supported corneal epithelial-cell adhesion, and the skirt supported stromal-cell adhesion [116].

Core–skirt architectures allocate optical, transport, and fixation requirements across spatially distinct regions [113]. OOKP provides durable biological anchorage through autologous tissue and requires complex tissue preparation and long-term clinical management [114,115]. Hydrogel-based systems provide regional control of optical clarity, mass transport, and cell-interactive properties [116]. Core–skirt interface stability, reproducible regional fabrication, nutrient transport, and stable skirt integration are central requirements for long-term performance [117].

### 4.3. Particle-Based Therapeutic and Reinforcement Systems

Particle-containing composite systems are applied to ocular-surface and stromal defects associated with inflammation, infection, or neovascularization. Drug carriers and functional particles provide local sustained delivery, antibacterial activity, anti-inflammatory effects, and antiangiogenic activity. Some particle phases also reinforce the surrounding polymer matrix. Key evaluation endpoints include therapeutic efficacy, release behavior, ocular safety, optical clarity, and effects on construct stability [118].

Drug-loaded microspheres can prolong local drug exposure and regulate inflammation and neovascularization. Minocycline-loaded nano-hydroxyapatite/poly(lactic-co-glycolic acid) (nHAP/PLGA) microspheres provide sustained release, and nHAP buffers acidic products generated during PLGA degradation. In an alkali-burn model, this system inhibited corneal neovascularization and promoted epithelial healing without evident cytotoxicity [119]. PLGA microspheres loaded with the antimicrobial peptide OH-CATH30 showed an initial release phase followed by sustained release in a bacterial keratitis model, with antibacterial activity and ocular tolerance [120].

Functional nanoparticles can provide anti-infective, anti-inflammatory, and antiangiogenic activity. Gelatin-capped silver nanoparticles showed antibacterial and antiangiogenic effects in a Staphylococcus aureus keratitis model. Local tolerance and long-term ocular safety remain important considerations [121]. A nanocellulose-reinforced, dexamethasone-loaded collagen hydrogel maintained optical clarity, improved resistance to enzymatic degradation, and provided sustained anti-inflammatory activity. This design combined structural reinforcement with local therapeutic delivery [122].

Particle-containing systems are appropriate for inflammatory, infectious, or neovascularized defects that require local therapy together with ocular-surface coverage or stromal support [118]. Drug-loaded microspheres provide controlled local delivery, functional nanoparticles add antimicrobial or antiangiogenic activity, and nanocellulose-containing systems can also improve structural stability. Current evidence is derived mainly from in vitro and animal studies [119,120,121,122]. Long-term ocular safety, optical performance, and clinical benefit require further investigation. Table 3 summarizes hybrid and composite functional polymer systems for corneal repair.

## 5. Conclusions and Perspectives

Corneal polymer design should be guided by the affected corneal layer, defect type, required functional duration, and intended repair outcome. Natural polymers provide tissue-relevant biological cues but vary in mechanical performance and batch consistency. Synthetic polymers offer defined composition, tunable properties, and reproducible processing but often require surface modification or ligand incorporation to improve cell interaction. Composite constructs assign optical, structural, and therapeutic functions to different components or regions, making interfacial stability and fabrication control central design requirements.

Current clinical evidence supports amniotic-membrane-supported limbal epithelial sheets for epithelial replacement in limbal stem cell deficiency [79], although limited cell availability and incomplete long-term barrier restoration restrict wider use. For stromal repair, collagen-based implants have been evaluated in humans [23], and BPCDX has been used for intrastromal augmentation in advanced keratoconus [27]. These constructs must preserve corneal curvature, transparency, and biomechanical support during tissue remodeling. No standalone substitute is established for Bowman’s layer; thin collagen-based interfaces remain experimental.

For Descemet’s membrane support and endothelial cell delivery, thin silk fibroin films have been evaluated experimentally as cell carriers [43,44,45]. Clinical use requires reliable insertion, sustained endothelial cell density, intact barrier function, and adequate fluid pumping. For full-thickness optical replacement, PMMA-based keratoprostheses provide an established clinical reference. Their principal long-term complications include device retention failure, tissue melting, retroprosthetic membrane formation, infection, and glaucoma [86,87,88].

The following quantitative values provide formulation-specific benchmarks rather than a direct material ranking because the studies used different specimens, formulations, and test conditions. Ex vivo human corneas showed 87.1% central transmittance at 500 nm after normalization to a thickness of 550 μm [3]. A compressed-collagen/laser-perforated PLGA construct reached a maximum tensile stress of 3.42 MPa, while its transmittance at 500 nm increased from 63% to 72% after 7 days in phosphate-buffered saline [108]. PCL/G-GMA reinforcement increased suture-tear resistance from approximately 0.15 N for the hydrogel alone to 4.32 N, compared with 7.45 N for human cornea, and more than 40% of the construct remained after 36 h in concentrated collagenase [109]. Incorporation of pHEMA into GelMA increased the compressive modulus from 6.53 to 155.49 kPa and produced approximately 90% transmittance at 700 nm [111]. In a post-LASEK comparison, the silicone hydrogel bandage lens had an oxygen transmissibility (Dk/t) of 99 × 10^−9^ barrer/mm, compared with 26.7 × 10^−9^ barrer/mm for the conventional hydrogel lens [97]. Table 4 summarizes the relative priority of the five design-requirement domains for the major corneal indications.

Material evaluation should integrate optical performance, mechanical performance and surgical handling, mass transport and degradation, biological integration, and manufacturability. Increasing crosslink density or fiber reinforcement improves structural stability but may restrict diffusion, reduce optical uniformity, or alter tissue remodeling. Drug incorporation introduces additional requirements for dose control, release kinetics, local exposure, and ocular safety.

Clinical translation requires reproducible manufacturing and indication-specific long-term evidence. Reports should specify material composition, fabrication conditions, sterilization method, storage stability, and batch-to-batch variation. Preclinical studies should measure tissue integration, degradation or drug release, inflammatory responses, long-term structural stability, visual performance, and innervation when the intended application requires nerve regeneration. Clinical studies should use indication-specific endpoints, longer follow-up, standardized evaluation methods, and direct comparison with established treatments.

## Figures and Tables

**Table 1:** Natural functional polymer systems and tissue-derived extracellular matrix comparators for corneal repair, organized by clinical indication and design requirements.

Material System	Corneal Indication	Polymer Design and Processing	Optical Performance	Mechanical Performance and Surgical Handling	Mass Transport and Degradation Behavior	Biological Function and Tissue Integration	Manufacturing and Translational Consideration	References
Collagen	Stromal replacement and defect repair (In vitro, animal, and clinical)	Fibril organization, crosslink chemistry, and in situ gelation	High when fibril diameter and spacing are controlled	Native gels are weak, and crosslinking improves integrity and handling	Hydrated and enzyme degradable, with crosslinking influencing transport and remodeling	ECM-related cues and cell interaction	Source/purification/batch control; curvature and remodeling support	[18,19,20,24,25,26,27,28]
RHC-III	Biosynthetic stromal implants and intrastromal repair (Clinical)	Defined recombinant sequence, formulation, and crosslinking	High and formulation dependent	Crosslinking controls stability, while suture retention can remain limited	Hydrated network with controllable enzymatic degradation	Host epithelial, stromal cell, and nerve ingrowth	Scale-up; cost; curvature and remodeling support	[21,22,23,30]
Gelatin and GelMA	Stromal bioinks, bioadhesives, and defect repair (In vitro and animal)	Degree of methacrylation, polymer concentration, photoinitiator formulation, and light dose	High in optically uniform networks; ~78% at 700 nm	Network density controls stiffness, handling, and print fidelity	Hydration and mesh size regulate diffusion and degradation	Cell binding motifs support cell adhesion	Photoinitiator safety; sterilization; formulation reproducibility	[31,32,33,34,35,36,37]
Silk fibroin	Epithelial and endothelial cell carriers and stromal scaffolds (In vitro and animal)	Degumming, beta sheet crystallinity, porosity, surface patterning, and multilayer assembly	High in thin films and controlled architectures; non-mulberry films (~15 μm), >95% in visible light	Crystallinity improves stability and scaffold handling	Porosity and crystallinity regulate transport and degradation	Supports epithelial, stromal, and endothelial cells	Source/sericin control; interface stability; endothelial handling and function	[38,41,42,43,44,45,46,47,48,49,50,51]
Chitosan	Ocular surface repair and endothelial cell delivery (In vitro)	Degree of deacetylation, molecular weight, film formation, and surface modification	Transparent in thin films	Film forming, with limited load bearing capacity	Hydrophilic and degradable	Antibacterial activity with limited intrinsic cell adhesion	Surface bioactivity; optical and process preservation	[52,53,54,55]
Hyaluronic acid	Ocular surface healing, defect repair, and cell delivery (In vitro and animal)	Molecular weight, methacrylation or dynamic crosslinking, and injectable processing	Generally high	Soft networks, with crosslinking providing retention and handling	High hydration with tunable residence time and degradation	Supports epithelial migration and inflammatory modulation	Crosslinking control; stability-transport balance; reproducibility	[56,57,58,59,60,61,62]
Alginate	Stromal bioinks and cell encapsulation (In vitro)	Guluronic acid content, calcium crosslinking, and secondary modification	Formulation dependent	Mild ionic gelation, with limited structural persistence	High hydration, ion exchange, and variable degradation	High early cell viability with limited cell adhesion	Retention; cell interaction; print reproducibility	[63,64,65]
Oxidized dextran	Injectable repair for alkali burn injury (In vitro and animal)	Oxidation degree and crosslinking with gelatin	Potentially clear in uniformly crosslinked networks	Injectable and formulation dependent	Tunable degradation	Supports preclinical wound healing	Long-term optical and tissue outcomes	[66]
Bacterial cellulose	Corneal dressings and epithelial repair (In vitro)	Biosynthesis, purification, and membrane processing	Good in thin films	Robust membrane with limited shape control	Slow degradation	Provides epithelial protection	Sterilization; storage stability; shape control	[67]
APCS	Lamellar keratoplasty and stromal replacement (Clinical)	Decellularization, preservation, and crosslinking	Native like stromal architecture	Good graft handling after appropriate processing	Native like diffusion with process-dependent degradation	Native ECM cues and stromal architecture	Tissue source; preservation; calcification risk	[68,69,70,71,72,73]
Amniotic membrane	Ocular surface reconstruction and limbal stem cell therapy (Clinical)	Tissue sourcing, preservation, and graft preparation	Optical performance is secondary to surface coverage and epithelial repair	Thin and non-load bearing	Variable persistence after implantation	Supports epithelial healing and inflammation modulation	Source/preservation consistency; cell supply; durable barrier restoration	[74,75,76,77,78,79,80]
Corneal dECM bioink	Stromal engineering and bioprinting (In vitro and animal)	Decellularization, matrix processing, formulation, and printing	Optical uniformity depends on formulation	Requires formulation optimization for structural stability	Formulation dependent	Provides cornea-specific matrix cues	Batch control; printing consistency	[81]

Note: Evidence refers to the cited formulation and indication. Clinical device use is not evidence of tissue regeneration. Native-cornea reference: 87.1 ± 2.0% at 500 nm, normalized to 550 μm thickness and near-physiological hydration [3]. Optical values are study-specific; hydrated thickness and measurement medium are not consistently specified in this review. No preferred standalone Bowman’s-layer substitute is established. Abbreviations: APCS, acellular porcine corneal stroma; dECM, decellularized extracellular matrix; ECM, extracellular matrix; GelMA, gelatin methacryloyl; RHC-III, recombinant human type III collagen.

**Table 2:** Synthetic functional polymer systems for corneal repair, organized by clinical indication and design requirements.

Material System	Corneal Indication	Polymer Design and Processing	Optical Performance	Mechanical Performance and Surgical Handling	Mass Transport and Degradation Behavior	Biological Function and Tissue Integration	Manufacturing and Translational Consideration	References
PCL	Fibrous scaffolds for stromal engineering (In vitro)	Molecular weight, fiber alignment, electrospinning, and melt electrowriting	Fibers may cause light scattering	Aligned fibers provide durable support and handling	Porosity controls transport and slow resorption	Topographic guidance for keratocytes	Fiber diameter; degradation rate	[89,90,91]
PLGA	Temporary epithelial and endothelial delivery (In vitro)	Lactide glycolide ratio, molecular weight, and porous or fibrous processing	Limited in fibrous formats	Temporary structural support	Tunable degradation with acidic degradation products	Surface modification supports cell interaction	Degradation profile; local tolerance	[92,93,94]
PGS	Exploratory elastic support for corneal engineering (In vitro)	Prepolymer chemistry, curing conditions, and molding followed by thermal curing.	Direct corneal optical data remain limited	Elastic support with surface erosion behavior	Degradable elastomer with surface erosion	Direct corneal evidence remains limited	Process control; long-term corneal compatibility	[95]
PHEMA	Core–skirt keratoprostheses (Clinical)	Crosslinking, hydration, and porous skirt architecture	Transparent central optic	Stable hydrated network and porous skirt for fixation	Hydrated and nondegradable	Peripheral tissue ingrowth through porous regions	Long-term retention; deposits; calcification; stromal melt	[83,84,85,96]
Silicone hydrogels	Bandage contact lenses and ocular surface protection (Clinical)	Lens processing, water content, and surface treatment	Transparent	Soft and flexible	Nondegradable hydrated network with high oxygen transmissibility	Supports short term epithelial protection	Tear-film stability; friction; infection; wear safety	[97,98,99]
PVA	Contact lenses and exploratory stromal membranes (In vitro and animal)	Polymer concentration and freeze–thaw physical crosslinking	High clarity in suitable formulations	Tough and elastic physical network	Hydration and diffusion decrease with repeated freeze–thaw cycles	Generally cytocompatible	Long-term corneal performance; processing consistency	[100,101,102]
PEG	Injectable repair matrices and cell delivery platforms (In vitro)	Molecular weight, crosslink chemistry, biofunctionalization, and injectable formulation	Generally transparent	Network design controls mechanical behavior	Crosslink chemistry governs persistence or degradation	Limited intrinsic cell interaction without modification	Surface bioactivity; sterilization; formulation reproducibility	[103]
PMMA	Central optics for full-thickness keratoprostheses (Clinical)	Precision machining, compression molding, and injection molding	High optical stability	Rigid, durable optical component	Low hydration and limited aqueous nutrient transport	Limited intrinsic tissue interaction	Retention; glaucoma; retroprosthetic membrane; melt; infection; lifelong follow-up	[86,87,88]
PDMS	Ocular surface interfaces and keratoprosthesis-related devices (In vitro)	Siloxane chemistry, surface treatment, and substrate stiffness	Transparent and oxygen permeable	Flexible and compliant interface	Limited aqueous solute transport and stable nondegradable behavior	Surface hydrophobicity limits cell adhesion and tear film spreading	Surface modification; interface stability	[104,105]

Note: Evidence refers to the cited formulation and indication. Human lens or keratoprosthesis experience does not establish regenerative efficacy. Qualitative optical descriptions are retained where comparable wavelength-specific data and hydrated thickness are unavailable in the reviewed account. Abbreviations: PCL, poly(caprolactone). PLGA, poly(lactic-co-glycolic acid). PGS, poly(glycerol sebacate). PHEMA, poly(2-hydroxyethyl methacrylate). PVA, poly(vinyl alcohol). PEG, poly(ethylene glycol). PMMA, poly(methyl methacrylate). PDMS, poly(dimethylsiloxane).

**Table 3:** Hybrid and composite functional polymer systems for corneal repair, organized by clinical indication and design requirements.

Material System	Corneal Indication	Polymer Design and Processing	Optical Performance	Mechanical Performance and Surgical Handling	Mass Transport and Degradation Behavior	Biological Function and Tissue Integration	Manufacturing and Translational Consideration	References
PCL and silk fibroin	Stromal regeneration (In vitro)	Component ratio, fiber diameter, alignment, and electrospinning	Fiber size dependent	Aligned fibrous support	Porosity-dependent transport and slow PCL resorption	Guides keratocyte attachment	Scattering; phase compatibility	[107]
Compressed collagen and perforated PLGA	Stromal and ocular surface repair (In vitro)	Plastic compression, membrane thickness, and laser perforation	63 ± 2.17% (0 d), 72 ± 1.8% (7 d) at 500 nm	Improved handling and suture retention	Perforations support diffusion and PLGA degrades	Collagen supports cell attachment	Multilayer interface stability; reproducible perforation	[108]
PCL microfiber and G-GMA hydrogel	Stromal repair (In vitro)	Microfiber spacing, fiber alignment, hydrogel formulation, and infusion	Transparent G-GMA core; non-transparent fiber-reinforced skirt	Reinforced and suturable	Hydrogel mediated transport	Supports corneal cell migration	Fiber-related scattering; component integration	[109]
GelMA and PEGDA	Anterior lamellar repair (In vitro and animal)	Polymer ratio, photocuring conditions, and DLP printing	PEGDA–GelMA-10-5, -15-5, and -20-5 was 90.7%, 86.3%, and 82.5% at 600 nm	Enhanced stability and print fidelity	Hydrated network with formulation-dependent transport and degradation	Supports epithelial and stromal repair	Crosslinking and interlayer control; photoinitiator safety	[110]
GelMA and PHEMA IPN	Stromal model (In vitro)	Interpenetrating network formation and network density	High optical clarity	Higher compressive modulus	Hydrated network with density-dependent transport	Maintains keratocyte viability and phenotype	Network density; transport-fabrication balance	[111]
GelMA-incorporated decellularized corneal matrix	Stromal reconstruction (In vitro)	GelMA impregnation and in situ photocrosslinking within decellularized corneal matrix	Optical uniformity depends on dECM content	Mechanical performance depends on matrix integrity and crosslinking	Formulation-dependent degradation	Provides cornea-specific ECM cues	Batch control; matrix consistency; optical uniformity	[112]
PHEMA core–skirt keratoprosthesis	full-thickness keratoprosthesis (Animal)	Integrated PHEMA core–skirt fabrication and porous skirt design	Transparent central optic	Porous skirt improves fixation	Hydrated nondegradable network	Fibrovascular ingrowth at the skirt	Core–skirt interface stability; melt; deposits; retention	[14,113]
OOKP clinical comparator	High risk full-thickness keratoprosthesis (Clinical)	PMMA optic combined with tooth bone and oral mucosa through surgical assembly	Transparent PMMA central optic	Durable biological anchorage	Device-dependent transport	Autologous tissue fixation	Surgical complexity; glaucoma; mucosal complications; comparator-only evidence	[114,115]
Collagen-functionalized PEG/PAA core and PHEA skirt	Hydrogel keratoprosthesis (In vitro)	Photolithographic regional fabrication and regional polymer selection	Transparent double network core	Region-specific support	Hydrated nutrient permeable design	Type I collagen on both core and skirt surfaces supports epithelial and stromal-cell adhesion	Regional fabrication; interface stability; process reproducibility	[116]
Minocycline loaded nHAP PLGA microspheres	Alkali burn neovascularization (In vitro and animal)	Microsphere composition, drug loading, particle size, and encapsulation	Corneal opacity was assessed using an in vivo alkali-burn model	No primary mechanical role	Sustained release with nHAP buffering of PLGA acidity	Antiangiogenic activity and epithelial healing support	Particle size; burst release; retention; distribution/clearance; ocular tolerance	[119]
OH CATH30 PLGA microspheres	Bacterial keratitis (In vitro and animal)	Peptide loading, microsphere size, and encapsulation	Corneal clinical signs were assessed in rabbit eyes	No primary mechanical role	Initial release followed by sustained release	Antibacterial activity and ocular tolerance	Peptide loading; burst release; residence; sterilization; pharmacokinetics; repeated-dose safety	[120]
Gelatin capped AgNPs	Bacterial keratitis (In vitro and animal)	Nanoparticle size, gelatin coating, loading, and dispersion	May increase light scattering	No primary mechanical role	Silver ion mediated release	Antibacterial and antiangiogenic activity	Dispersion; migration/clearance; burst release; chronic toxicity; optical haze	[121]
Nanocellulose dexamethasone collagen	Inflammatory corneal defects (In vitro and animal)	Nanocellulose content, drug loading, collagen crosslinking, and particle dispersion	Maintains clear transparency	Improved resistance to enzymatic degradation	Sustained drug release	Anti-inflammatory activity	Dispersion; ocular safety; batch consistency; migration/clearance; optical haze	[122]

Note: Evidence refers to the specific construct. OOKP is included as a clinical comparator, whereas the PHEMA construct is supported by evidence from rabbit studies. Optical values are formulation-specific and have not been normalized for cross-study comparison; hydrated thickness and measurement medium should therefore be considered when interpreting transmittance. Abbreviations: PCL, poly(caprolactone); PLGA, poly(lactic-co-glycolic acid); G-GMA, gelatin glycidyl methacrylate; GelMA, gelatin methacryloyl; PEGDA, poly(ethylene glycol) diacrylate; DLP, digital light processing; IPN, interpenetrating polymer network; dECM, decellularized extracellular matrix; OOKP, osteo-odonto-keratoprosthesis; PMMA, poly(methyl methacrylate); PHEMA, poly(2-hydroxyethyl methacrylate); PEG, poly(ethylene glycol); PAA, poly(acrylic acid); PHEA, poly(2-hydroxyethyl acrylate); nHAP, nano-hydroxyapatite; AgNPs, silver nanoparticles.

**Table 4:** Indication-guided priority matrix across five design-requirement domains.

Clinical Indication	Optical Performance	Mechanical Performance and Handling	Mass Transport and Degradation	Biological Function and Integration	Manufacturing and Translation	References
Ocular surface and epithelium	Medium, functional visual clarity	Medium, placement and retention	Medium, tear and nutrient exchange	High, epithelial healing and inflammation control	Medium, reproducible topical formats	[10,59,60,66,74,75,76,77,78,79,80,97,98,99]
Stromal repair	High, transparency and refractive quality	High, load bearing and suture support	Medium, nutrient diffusion and remodeling	Medium, stromal integration and quiescence	Medium, shape and thickness control	[18,19,20,21,22,23,24,25,26,27,28,29,30,34,35,36,37,38,39,40,41,42,54,62,63,64,65,66,67,68,69,70,71,72,73,81,107,108,109,110,111,112]
Endothelial delivery	High, optical clarity	Medium, insertion and stable positioning	High, oxygen and glucose exchange	High, endothelial phenotype and pump function	Medium, reproducible ultrathin carriers	[12,13,43,44,45,55,92]
Full-thickness replacement	High, central transparency	High, pressure resistance and surgical fixation	Medium, nutrient diffusion and durability	Medium, epithelialization and host integration	High, scalable multilayer fabrication	[14,83,84,85,86,87,88,96,113,114,115,116,117]
Therapeutic defects	Low, temporary optical demand	Low, local retention only	High, release and degradation control	High, anti-infective or anti-inflammatory action	Medium, dose and batch consistency	[66,118,119,120,121,122]

Note, High indicates a primary requirement, Medium indicates an important supporting requirement, and Low indicates a lower relative priority for the specified indication. All five domains remain relevant.

## Data Availability

No new data were created or analyzed in this study. Data sharing is not applicable to this article.:

## Abbreviations

The following abbreviations are used in this manuscript:

AgNPs	silver nanoparticles
AM	amniotic membrane
APCS	acellular porcine corneal stroma
BPCDX	double-crosslinked bioengineered porcine collagen construct
CD44	cluster of differentiation 44
dECM	decellularized extracellular matrix
DLP	digital light processing
DMEK	Descemet membrane endothelial keratoplasty
DN	double-network
DOPA	3,4-dihydroxyphenylalanine
ECM	extracellular matrix
EDC	1-ethyl-3-(3-dimethylaminopropyl)carbodiimide
G-GMA	gelatin glycidyl methacrylate
GelMA	gelatin methacryloyl
HA	hyaluronic acid
IPN	interpenetrating polymer network
LAP	lithium phenyl-2,4,6-trimethylbenzoylphosphinate
LSCD	limbal stem cell deficiency
MPC	2-methacryloyloxyethyl phosphorylcholine
nHAP	nano-hydroxyapatite
NHS	N-hydroxysuccinimide
OOKP	osteo-odonto-keratoprosthesis
PAA	poly(acrylic acid)
PCL	poly(caprolactone)
PDMS	poly(dimethylsiloxane)
PEG	poly(ethylene glycol)
PEGDA	poly(ethylene glycol) diacrylate
PEGDA-DOPA	DOPA-modified poly(ethylene glycol) diacrylate
PGS	poly(glycerol sebacate)
PHEA	poly(2-hydroxyethyl acrylate)
PHEMA	poly(2-hydroxyethyl methacrylate)
PLGA	poly(lactic-co-glycolic acid)
PMMA	poly(methyl methacrylate)
PVA	poly(vinyl alcohol)
RGD	arginine–glycine–aspartic acid
RHC	recombinant human collagen
RHC-III	recombinant human type III collagen
SF	silk fibroin
UV	ultraviolet light
